# Developing a Centralized Hub for Research Data Services: Trainings and Resources in Health Sciences Contexts

**DOI:** 10.7191/jeslib.642

**Published:** 2023-02-16

**Authors:** Peace Ossom Williamson

**Affiliations:** NNLM National Center for Data Services, NYU Langone Health, New York, NY, US

## Abstract

**Objectives:**

In 2021, a new national center was funded by the National Library of Medicine (NLM) to build capacity for providing data services within health sciences libraries by coordinating, developing, and disseminating trainings, curricular resources, and curated pathways for learning. Research data services has been a growing service component for health sciences libraries for over a decade, and efforts have come from individuals, professional societies, task forces, and interest groups; however, there is still a great deal of unrealized potential in this area as well as growing needs driven by new requirements from funders and publishers and increasing demand from institutions for data science skills and support for data-related research needs.

**Methods:**

The National Center for Data Services (NCDS) was established in July 2021 and is the newest of the Network of the National Library of Medicine (NNLM)’s national offices and centers. NCDS works with the NNLM’s seven regional medical libraries and national offices and centers, and draws upon extensive connections with national data and library organizations, all toward the aim of developing capacity for data services in the health information community while centering efforts toward equity, diversity, and accessibility throughout.

## Developing a Centralized Hub for Research Data Services

As health sciences libraries continue to provide services, collections, and expertise around research data services, there is growing demand for resources and opportunities for learning to build skills in this area. Research data services (RDS) in libraries span a wide array of offerings, including supporting data access and reuse, data management and storage, data analysis and computing, and data curation and long-term preservation. These areas of services require a variety of skills, infrastructure, policies, and specialized knowledge that must be regularly updated due to a frequently changing landscape and new updates in technology. Furthermore, there is a growing need to support new requirements from publishers, institutions, and funders, particularly the new National Institutes of Health (NIH) requirements for data management and sharing that took effect in January 2023 (National Institutes of Health 2020). While a number of individuals, groups, and organizations have had focused efforts to build the capacity for providing data services within health sciences libraries, these efforts resulted in a landscape that could be difficult to navigate while gaps and unnecessary redundancies remained.

## Establishment of the NNLM National Center for Data Services

The Network of the National Library of Medicine (NNLM), established originally as the Regional Medical Library Program, has engaged in outreach efforts for the National Library of Medicine (NLM), promoting resources and health information literacy, and providing tools, training, and funding to advance NIH aims since 1965 (Speaker 2018). The National Center for Data Services (NCDS) was established at NYU Health Sciences Library, NYU Langone Health through a cooperative agreement between the NLM and Lamar Soutter Library, University of Massachusetts Chan Medical School in July 2021 to support the NLM’s strategic goals to accelerate discovery and advance health by building a workforce and providing the tools for data-driven research and healthcare (National Library of Medicine 2017). The focus is on knowledge application and consolidation through courses with coaching and opportunities to practice learned skills, with efforts toward equity, diversity, and accessibility as central to this effort. The NCDS integrates values of open science, reproducibility, inclusivity, and ethical use of data in an innovative educational portfolio while using a coordinated, data-driven approach to building capacity for data services among health information professionals. NCDS also draws on the support from the NYU Health Sciences Library Data Services Team as content experts with advanced subject knowledge.

### Trainings

NCDS develops new trainings and educational resources but also looks to careful curation of existing content and leveraging partnerships with other groups building resources rather than “reinventing the wheel.” Curricula developed within the NNLM fall under the scope of providing instruction on data literacy, research data management, and open science to support librarians and health information professionals in developing or advancing data services at their institutions. The main focus of the curriculum is to foster and encourage a data-driven workforce capable of advancing health through biomedical data science. The focus is on the following four outcomes for participants to be able to (1) provide expertise in policies and practices supporting open science and data sharing; (2) use NIH & NLM resources and common tools for data discovery and re/use; (3) analyze, evaluate, communicate, and visualize data; and 4) recommend or apply ethical practices in data science.

Trainings and resources are also developed to meet needs identified by the regional outreach units who work directly with health information professionals, health professions, and the public in regions that reflect needs distinct from common focuses (e.g., tribal colleges and historically Black colleges and universities). Through these partnerships, this organization is in a position to serve a national audience while tailoring offerings to the needs of individual communities, in particular, underserved communities.

These offerings are also informed by current research in equity and inclusion and principles of accessibility. Instructors, mentors, examples, and activities reflect diverse perspectives with emphasis on underrepresented voices and stories. In particular, considerations in data ethics serve as a guiding principle for all resources so that, for example, trainings in data science skills are accompanied by communication around the existence, effects, and potential mitigation strategies of bias and inequities in data. Similarly, trainings on data management and sharing include discussion of issues around sensitive data and data ownership.

### General Data Resources

NCDS has and continues to develop online resources for point of need.

### NNLM Data Glossary

The NCDS has developed an updated dictionary of RDS-related terms, such as persistent unique identifier, data lake, and data use agreement, in the NNLM Data Glossary. Visitors to the Data Glossary can browse or search for terms and access related information, including examples, tools, and further resources.

### Data Videos

Training recordings, video tutorials, and informational videos are provided on the NNLM YouTube channel in the NCDS Playlist. These include videos such as an overview of the NIH Data Management and Sharing Policy and an interview with three data librarians talking about their roles and backgrounds.

### Continuing Education Pathway

NCDS is also developing a website for navigating synchronous and asynchronous offerings for developing data skills and services with a pathway toward the various areas in order to show how the various offerings within and outside of NNLM align and build upon one another toward attainment of each goal.

### NIH Data Management and Sharing Policy Resources

NCDS has partnered with other organizations to develop a comprehensive toolkit of resources for health information professionals supporting their institutions as they work to meet the expectations of the incoming policy. These resources are developed in an extensive collaboration of workgroups consisting of members from the Medical Library Association Data Caucus, Data Curation Network, Research Data Access & Preservation Association, DMPTool, Data Discovery Collaboration, and other health and data librarians.

### For Librarians

Librarians becoming familiar with the policy can make use of a glossary of terminology in the DMSP and a policy readiness checklist for librarians preparing themselves and their institution for the policy. Librarians teaching and informing their communities about the policy can duplicate and edit the template libguide and slides.

### For Researchers

Researchers composing grant proposals can utilize the DMSP checklist for constructing a data management plan and the rubric for evaluating their plan prior submission.

### For Everyone

Examples of data management plans at varying levels of quality and guidance published in DMPTool can be used as a teaching aid or for researchers who are trying to get started or assess their own work. Furthermore, the repository finder interface creates an open-source and adaptable tool for finding repositories that meet NIH requirements.

Just as the NCDS has developed resources focused for a major area of need in 2021 and 2022, the specialized resources will continue to address current trends and areas of needs going forward.

### Summer Data Internship

In addition to the trainings and resources open to all, NCDS has a paid summer internship program which provides data training, project experience, and professional networks for LIS students from historically excluded racial and ethnic groups. The approximately 10-week summer internship partners interns with libraries who have concrete, completable data projects. The students present at an internal showcase at the end of the program and are encouraged to submit their work to a conference. The aim of the internship is to provide LIS students (and other graduate students of color in health sciences programs who may be interested in librarianship) with an introduction to data librarianship while building skills in project management and completion as well as presentation skills. Through this internship of mentored learning, interns are embedded into the tasks and complexities of data librarianship, while being provided ongoing guidance for success. Ultimately, they take away awareness and recognition of whether they are interested in data librarianship, and they have practical experience for seeking employment in the field, as well as connections to librarians working in the field.

### Reception and Preliminary Evaluation of NCDS Offerings

NCDS has seen early engagement with its offerings. Registrations have come from all 50 states and at least 15 other countries. Trainings that have limited registrations have filled each time, sometimes before full promotion of the offering. Moreover, the engagement goes further than registrations: 78% of people who registered for presentations about NCDS attended live, and 67% of people who registered for NCDS-led trainings attended live, while the recordings have seen almost three times the number of initial attendees in views within 3–6 months. Likewise, training programs that required acceptance saw high participation: The *Ethical Considerations of Data* course had 79% participation from accepted participants and the *Fundamentals of Health Sciences Research Data Management* had 87%. The internship acceptance rate was 24% due to high application numbers, and eight of the nine interns completed the program.

NCDS has also been engaging in ongoing evaluation efforts, eliciting ratings and feedback from participants that will be shared in efforts toward transparency and replicability for similar educationally-focused programs and initiatives.

## Conclusions

Based upon the ongoing demand for offerings to further develop data capacity in health information professionals, the establishment of the NCDS meets a crucial need. The team plans to continue to focus on knowledge application and skill acquisition through live trainings, cohort-model courses, and information sharing through online resources as the field advances toward the open movement and inclusivity in research.
